# Realizing up-conversion fluorescence tuning in lanthanide-doped nanocrystals by femtosecond pulse shaping method

**DOI:** 10.1038/srep13337

**Published:** 2015-08-20

**Authors:** Shian Zhang, Yunhua Yao, Xu Shuwu, Pei Liu, Jingxin Ding, Tianqing Jia, Jianrong Qiu, Zhenrong Sun

**Affiliations:** 1 State Key Laboratory of Precision Spectroscopy, and Department of Physics, East China Normal University, Shanghai 200062, China; 2 State key Laboratory of Luminescent Materials and Devices, and Institute of Optical Communication Materials, South China University of Technology, Guangzhou 510640, China; 3 NYU-ECNU Institute of Physics at NYU Shanghai, Shanghai, 200062, China

## Abstract

The ability to tune color output of nanomaterials is very important for their applications in laser, optoelectronic device, color display and multiplexed biolabeling. Here we first propose a femtosecond pulse shaping technique to realize the up-conversion fluorescence tuning in lanthanide-doped nanocrystals dispersed in the glass. The multiple subpulse formation by a square phase modulation can create different excitation pathways for various up-conversion fluorescence generations. By properly controlling these excitation pathways, the multicolor up-conversion fluorescence can be finely tuned. This color tuning by the femtosecond pulse shaping technique is realized in single material by single-color laser field, which is highly desirable for further applications of the lanthanide-doped nanocrystals. This femtosecond pulse shaping technique opens an opportunity to tune the color output in the lanthanide-doped nanocrystals, which may bring a new revolution in the control of luminescence properties of nanomaterials.

Because of the unique optical properties, such as large Stokes shift, high resistance to optical blinking and sharp luminescence peak, the lanthanide-doped nanomaterials have attracted considerable attention in the past twenty years, and have been successfully applied in laser source^1^,^2^, fiber-optic communication^3^,^4^, light-emitting diode^5^, color display^6^,^7^,^8^,^9^, biolabeling^10^,^11^,^12^, and so on. It was noteworthy that the up-conversion (UC) fluorescence generation by converting the low-frequency stimulation into high-frequency emission through two-photon or multi-photon absorption can greatly extend the related applications of the lanthanide-doped nanomaterials, especially in biomedical field^13^,^14^,^15^,^16^. Usually, this UC technique utilized the near-infrared (NIR) laser to excite the luminescent nanomaterials rather than ultraviolet (UV) laser, and therefore can significantly minimize background autofluorescence, photobleaching and photodamage. Recently, the optical properties of the UC fluorescence in lanthanide-doped nanomaterials have become an important research topic^17^,^18^, and how to improve the UC luminescent efficiency or realize the UC multicolor tuning was always the main concern of the researchers^18^.

Realizing the UC fluorescence tuning of the lanthanide-doped nanomaterials is critical for their applications in various related fields, such as multicolor laser output, optoelectronic conversion, three-dimensional display and multiplexed biological imaging. So far, various schemes have been proposed to tune the UC fluorescence. Changing the material properties was considered as a commonly used method, such as varying lanthanide dopant^19^,^20^,^21^,^22^, host matrix^23^,^24^,^25^, and particle infrastructure (i.e., the size, morphology and core-shell structure of nanoparticles)^26^,^27^,^28^,^29^. Controlling the laser parameters was another method that is often employed, such as varying the excitation wavelength^30^,^31^, power density^32^,^33^, repetition rate and pulse duration^34^, or applying two-color laser field^35^. Furthermore, utilizing electric or magnetic field has also shown to be an available method to obtain the UC fluorescence tuning^36^,^37^,^38^. Here, we propose a new scheme to tune the UC fluorescence in lanthanide-doped nanocrystals by a femtosecond pulse shaping technique. We take the common Er^3+^-doped NaYF_4_ nanocrystals dispersed in the glass as our study example, and utilize a square phase modulation to realize the green and red UC fluorescence tuning. By this simple spectral phase modulation, the initial femtosecond laser pulse can be shaped into multiple subpulses with the controllable time separation and relative intensity. Such a shaped femtosecond laser pulse can affect and even change the excitation pathways of the UC fluorescence generation, and consequently realize the green and red UC fluorescence tuning.

## Experimental arrangement

**Figure 1:**
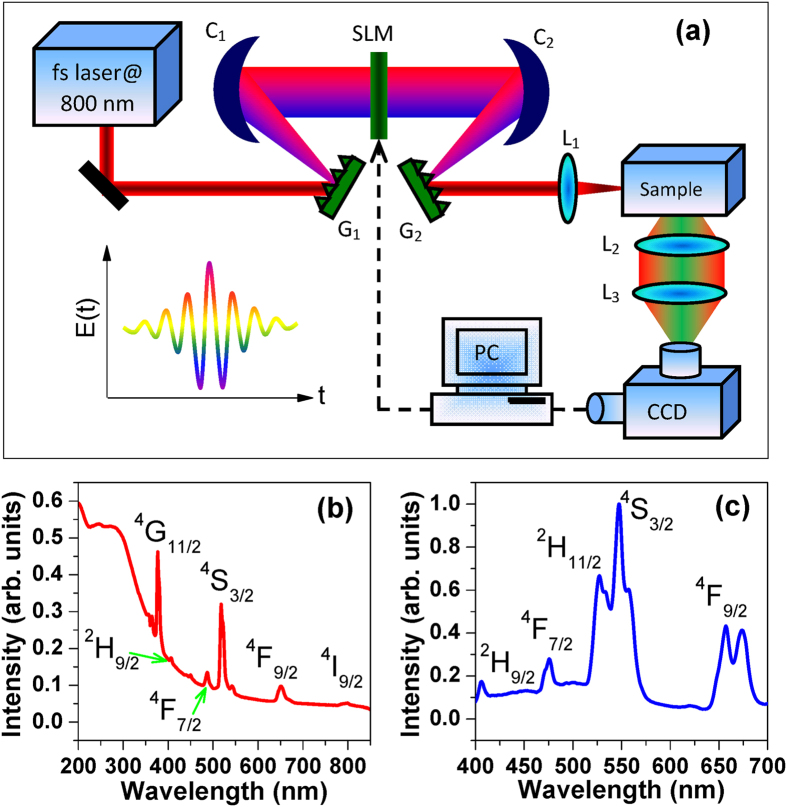
Experimental arrangement for controlling the UC fluorescence in lanthanide-doped nanocrystals by the femtosecond pulse shaping method (**a**), where G_1,2_ are diffraction grating, C_1,2_ are column concave mirror, SLM is spatial light modulator and L_1-3_ are optical lens, and the UV-VIS-NIR absorption spectrum (**b**) and the UC fluorescence spectrum in the visible light region (**c**) of 5%Er^3+^-doped NaYF_4_ nanocrystals.

Our experimental setup is schematically shown in Fig. 1(a). A Ti-sapphire mode-locked regenerative amplifier (Spectra-physics, Spitfire) is used as the excitation source with the pulse width of about 50 fs, central wavelength of 800 nm and repetition rate of 1 kHz. The output femtosecond laser pulse is sent into a programmable 4f configuration zero-dispersion pulse shaping system, which is composed of a pair of diffraction gratings with 1200 lines/mm (G_1_ and G_2_), a pair of concave mirrors with 200-mm focal length (C_1_ and C_2_) and an one-dimension liquid-crystal spatial light modulator (SLM-S320d, JENOPTIK), and the SLM is placed at the Fourier plane and used to vary the laser spectral phase and/or amplitude in the frequency domain. By properly designing the laser spectral phase and/or amplitude, it is possible to obtain such a shaped femtosecond laser pulse with almost arbitrary temporal distribution. The shaped femtosecond laser pulse is focused into the sample by a lens with 50-mm focal length (L_1_). All fluorescence signals emitted from the sample are perpendicularly collected by a telescope system (L_2_ and L_3_) and measured by a spectrometer with charge-coupled device (CCD).

We perform the experiment in 5%Er^3+^-doped NaYF_4_ nanocrystals dispersed in silicate glass with the composition of 40SiO_2_-25Al_2_O_3_-18NaCO_3_-10YF_3_-7NaF-5ErF_3_ (mol.%), here the sample is prepared by melt-quenching at 1450 °C for 45 minutes and successive heat treatment at 450 °C for 10 hours. The X-ray diffraction (XRD) pattern and transition electron microscopy (TEM) image have been demonstrated in our previous work^35^, which showed the existence of cubic α-NaYF_4_ crystalline phase and the homogeneous distribution of spherical nanocrystals with an average size of 20–30 nm. Figure 1(b) shows the UV-VIS-NIR absorption spectrum of Er^3+^-doped NaYF_4_ nanocrystals. One can see that six main absorption bands are observed around the wavelengths of 377, 407, 487, 518, 651 and 799 nm, which are corresponding to these excited states ^4^G_11/2_, ^2^H_9/2_, ^4^F_7/2_, ^4^S_3/2_, ^4^F_9/2_ and ^4^I_9/2_. In our experiment, the UC fluorescence spectrum in the visible light region is shown in Fig. 1(c). As can be seen, five fluorescence signals can be observed around the wavelengths of 408, 475, 527, 547 and 656 nm, which are attributed to the state transitions ^2^H_9/2_, ^4^F_7/2_, ^2^H_11/2_, ^4^S_3/2_, ^4^F_9/2_→^4^I_15/2_, respectively. It is obvious that the green and red UC fluorescence dominates the visible light spectrum, and therefore our main goal in this work is to tune the green and red UC fluorescence by shaping the femtosecond laser pulse with a spectral phase modulation.

**Figure 2:**
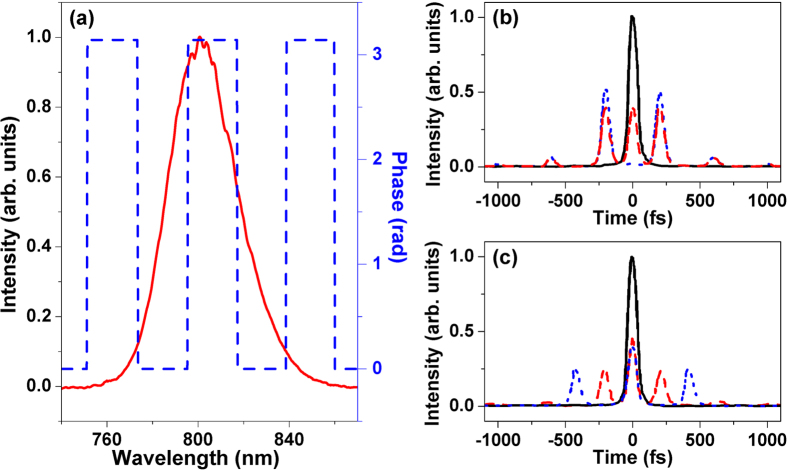
The shaped femtosecond laser spectrum by a square phase modulation in the frequency domain (**a**), and the temporal intensity distribution of the shaped femtosecond laser pulse for the modulation depths Δ = 0 (black solid line), 0.75π (red dashed line) and π (blue dotted line) with the modulation time Γ = 1062 fs (**b**) and for the modulation times Γ = 0 (black solid line), 1062 (red dashed line) and 2124 fs (blue dotted line) with the modulation depth Δ = 0.5π (**c**).

In our experiment, we utilize a square phase modulation to control the green and red UC fluorescence tuning of Er^3+^-doped NaYF_4_ nanocrystals, and the spectral phase modulation is schematically shown in Fig. 2(a). Mathematically, the square phase modulation can be defined by such a function of 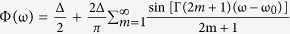
39, where Δ and Г respectively represent the modulation depth and time, ω_0_ is the laser central frequency, and m is the integral number. Figures. 2(b,c) present the temporal intensity distribution of the shaped femtosecond laser pulse for the modulation depths Δ = 0 (black solid line), 0.75π (red dashed line) and π (blue dotted line) with the modulation time Γ = 1062 fs and for the modulation times Γ = 0 (black solid line), 1062 (red dashed line) and 2124 fs (blue dotted line) with the modulation depth Δ = 0.5π, respectively. It is shown that the square phase modulation will lead to a sequence of subpulses with a controllable time separation and relative intensity. The modulation depth Δ is to control the relative intensity between the central subpulse and those side subpulses (see Fig. 2(b)), and the modulation time Г is to control the time separation of these subpulses (see Fig. 2(c)). Here, we fix the modulation depth Δ = π and vary the modulation time Г to control the green and red UC fluorescence intensities of Er^3+^-doped NaYF_4_ nanocrystals.

## Results and discussion

**Figure 3:**
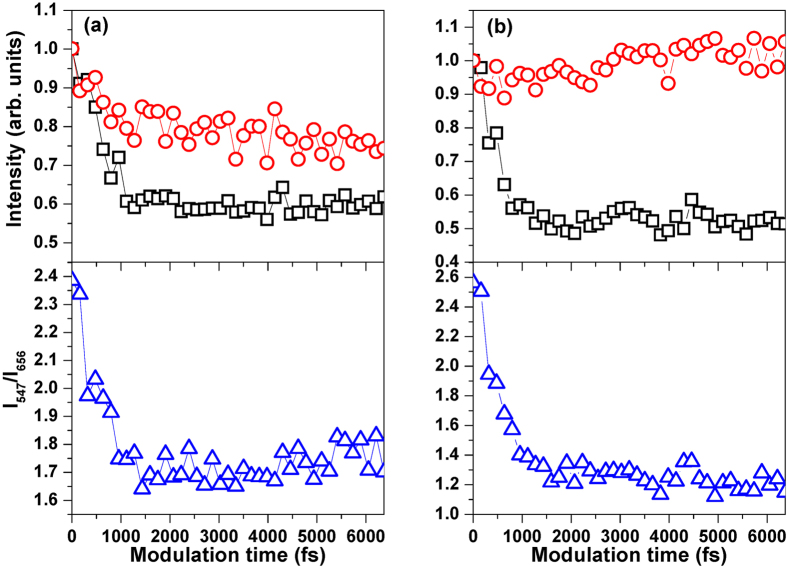
Normalized green and red UC fluorescence intensities at the wavelengths of 547 (black squares) and 656 nm (red circles) (I_547_ and I_656_) as the function of the modulation time Г with the laser intensities of 1.2 × 10^13^ (**a**) and 2.4 × 10^12^ W/cm^2^ (**b**) (upper panels), together with the fluorescence intensity ratio I_547_/I_656_ (lower panels).

Figure 3 shows the normalized green and red UC fluorescence intensities at the wavelengths of 547 (black squares) and 656 nm (red circles) (labeled with I_547_ and I_656_) as the function of the modulation time Г with the laser intensities of 1.2 × 10^13^ (a) and 2.4 × 10^12^ W/cm^2^ (b) (see upper panels). All experimental data are normalized by the unshaped femtosecond pulse excitation, and hereafter the same method is utilized. As can be seen, by varying the modulation time Г, both I_547_ and I_656_ can be efficiently suppressed in the higher laser intensity, but their control efficiencies are different, and I_547_ obtains the higher control efficiency (see Fig. 3(a)). However, I_656_ almost keeps unchanged and I_547_ is effectively suppressed in the lower laser intensity (see Fig. 3(b)). Here, the control efficiency is defined by the function of 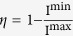, and I^max^ and I^min^ are the maximum and minimum fluorescence intensities, respectively. Since the control efficiencies of I_547_ and I_656_ are different by varying the modulation time Г, the green and red UC fluorescence tuning can be realized by this simple spectral phase modulation. Figure 3 also presents the fluorescence intensity ratio I_547_/I_656_ by varying the modulation time Г (see lower panels). It can be seen that the ratio I_547_/I_656_ can be controlled in the range of 1.7–2.4 in the higher laser intensity while 1.1–2.6 in the lower laser intensity. Obviously, the tuning range of the green and red UC fluorescence is higher in the lower laser intensity. Consequently, one can conclude that the square phase modulation can provide an alternative scheme to tune the green and red UC fluorescence in Er^3+^-doped NaYF_4_ nanocrystals.

**Figure 4:**
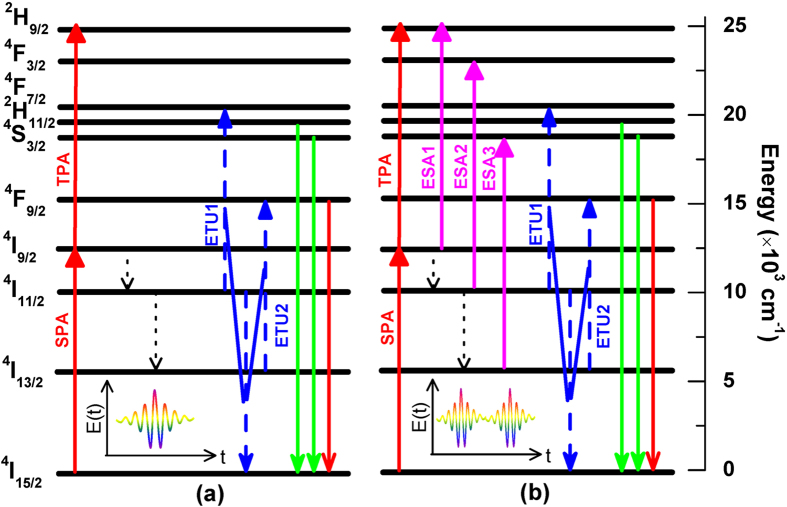
Energy level diagram of Er^3+^ ion and the possible UC processes for green and red fluorescence generation by the unshaped femtosecond laser pulse (**a**) and the shaped femtosecond laser pulse with the square phase modulation (**b**).

To illustrate the physical control process of the green and red UC fluorescence tuning by the square phase modulation in Fig. 3, we present the energy level diagram of Er^3+^ ion and the possible up-conversion processes for the green and red fluorescence generation by the unshaped femtosecond laser pulse and the shaped femtosecond laser pulse with the square phase modulation, as shown in Fig. 4. As can be seen in Fig. 4(a), since the laser pulse separation of 1 ms (corresponding to the laser repetition rate of 1 kHz) is far larger than the excited state lifetime of Er^3+^ ion in the range of microseconds in our experiment, only one laser pulse is excited within the excited state lifetime for the unshaped femtosecond laser pulse. The population in the ground state ^4^I_15/2_ is pumped to the excited state ^4^I_9/2_ by single-photon absorption (SPA) and the excited state ^2^H_9/2_ by resonance-mediated two-photon absorption (TPA). The population in the excited state ^2^H_9/2_ will also spontaneously decay to the three lower excited stats ^2^H_11/2_, ^4^S_3/2_ and ^4^F_9/2_, and then emits the green and red UC fluorescence. The population in the excited state ^4^I_9/2_ will spontaneously decay to the two lower excited states ^4^I_11/2_ and ^4^I_13/2_, and then can be further excited to the higher excited states ^4^F_7/2_ and ^4^F_9/2_ by two energy transfer up-conversion processes (ETU1 and ETU2). The dopant concentration determines the average distance between the neighboring dopant ions, and so the ETU process is only generated in the higher dopant concentration. Therefore, comparing with the green UC fluorescence, the red UC fluorescence generation additionally contains the ETU2 processes. As can be seen in Fig. 4(b), multiple subpulses are excited within the excited state lifetime since a sequence of subpulses are formed by the square phase modulation, and thus these excited state absorption (ESA) processes from the excited states ^4^I_9/2_ to ^2^H_9/2_, ^4^I_11/2_ to ^4^F_3/2_ and ^4^I_13/2_ to ^4^S_3/2_ can occur in addition to those excitation processes in Fig. 4(a), which are labeled with ESA1-3, respectively. The population in the excited states ^2^H_9/2_ and ^4^F_3/2_ by the ESA1,2 processes will also spontaneously decay to the three lower excited states ^2^H_11/2_, ^4^S_3/2_ and ^4^F_9/2_, and emits the green and red UC fluorescence. However, the population in the excited state ^4^S_3/2_ by the ESA3 process will directly emit the green UC fluorescence. In this case, the green UC fluorescence generation results from the TPA, ESA1-3 and ETU1 processes while the red UC fluorescence generation comes from the TPA, ESA1,2 and ETU1,2 processes.

Since the green and red UC fluorescence results from different excited pathways for the unshaped and shaped femtosecond laser pulses and even for the same femtosecond laser pulse shape, the green and red UC fluorescence tuning in Fig. 3 can be well explained by analyzing these TPA, ESA and ETU processes. The control efficiencies of the green and red UC fluorescence by the TPA, ESA1,2 and ETU1 processes should be the same because the population in the excited states ^2^H_11/2_, ^4^S_3/2_ and ^4^F_9/2_ comes from the spontaneous decay of the higher excited states ^2^H_9/2_, ^4^F_3/2_ and ^4^F_7/2_. However, the ESA3 process leads to the green UC fluorescence generation and therefore will suppress its control efficiency. Similarly, the ETU2 process leads to the red UC fluorescence generation and also will suppress its control efficiency. The weight difference of the ESA3 process in the green UC fluorescence generation and the ETU2 process in the red UC fluorescence generation results in their different control efficiencies by varying the modulation time Г of the square phase modulation.

**Figure 5:**
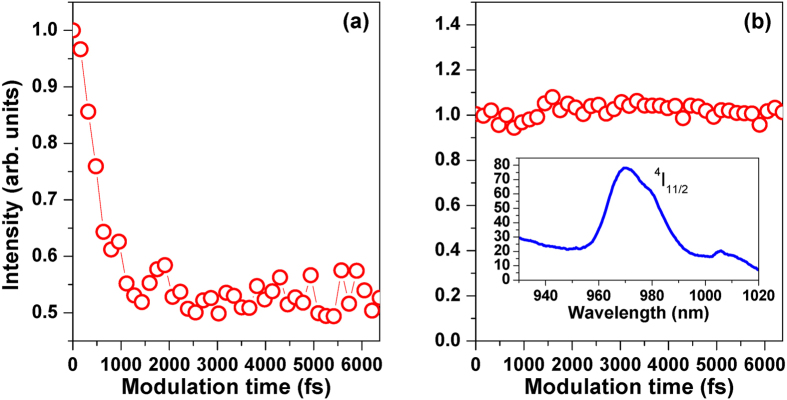
Normalized purple UC fluorescence intensity at the wavelength of 408 nm I_408_ (**a**) and near-infrared fluorescence intensity at the wavelength of 970 nm I_970_ (**b**) as the function of the modulation time Г with the laser intensity of 1.2 × 10^13^ W/cm^2^. Inset in Fig. 4(b) shows the near-infrared fluorescence spectrum.

To demonstrate that the ESA3 and ETU2 processes will respectively suppress the control efficiencies of the green and red UC fluorescence, we present the normalized purple UC fluorescence intensity at the wavelength of 408 nm (labeled with I_408_) by varying the modulation time Г with the laser intensity of 1.2 × 10^13^ W/cm^2^, as shown in Fig. 5(a). The purple UC fluorescence generation comes from the TPA and ESA1 processes, and therefore will not be affected by the ESA3 and ETU2 processes. One can see that the control efficiency of I_408_ (∼48%) is higher than that of I_547_ and I_656_ (∼40% and 25%), and such an experimental observation can well validate our above deduction. Figure 5(b) presents the normalized near-infrared fluorescence intensity at the wavelength of 970 nm (labeled with I_970_) by the square phase modulation, together with this fluorescence spectrum (see inset in Fig. 5(b)). This near-infrared fluorescence signal results from the state transition ^4^I_11/2_→^4^I_15/2_, and therefore will not be affected by the laser spectral phase, because the population in the excited state ^4^I_11/2_ comes from the spontaneous decay of the excited state ^4^I_9/2_, and the absorption in the excited state ^4^I_9/2_ is a SPA process, which is independent of the laser spectral phase. As expected, I_970_ keeps a constant. Similarly, since the ETU2 process is correlated with the two excited states ^4^I_11/2_ and ^4^I_13/2_, the red UC fluorescence component by the ETU2 process should be constant by the square phase modulation, which is different from the green UC fluorescence component by the ESA3 process.

**Figure 6:**
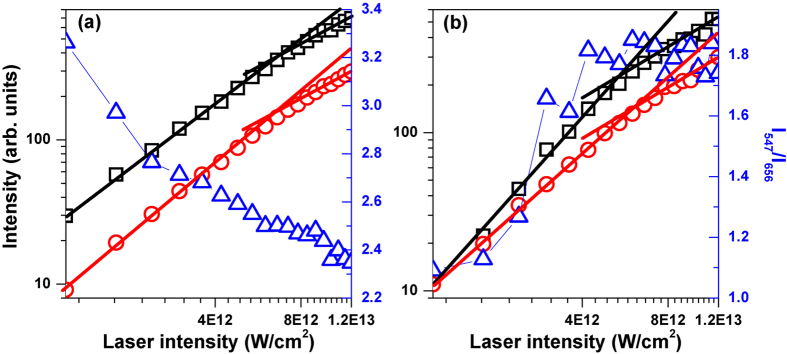
Green and red UC fluorescence intensities at the wavelengths of 547 (black squares) and 656 nm (red circles) (I_547_ and I_656_) by varying the laser intensity for the unshaped femtosecond laser pulse (**a**) and the shaped femtosecond laser pulse with the modulation time of Г = 6370 fs (**b**), together with the fluorescence intensity ratio I_547_/I_656_ (blue triangles). The two solid lines are used to linearly fit the experimental data in the low and high laser intensities, respectively.

Figure 6 shows the green and red UC fluorescence intensities I_547_ (black squares) and I_656_ (red circles) by varying the laser intensity for the unshaped femtosecond laser pulse (a) and the shaped femtosecond laser pulse with the modulation time of Г = 6370 fs (b), together with the fluorescence intensity ratio I_547_/I_656_ (blue triangles). In higher laser intensity (>6 × 10^12^ W/cm^2^), the UC fluorescence will be saturated, and so these experimental data in the low and high laser intensities are linearly fitted by two solid lines with different slopes, respectively. For the unshaped femtosecond laser pulse, the slope of the red UC fluorescence is slightly larger than that of the green UC fluorescence because of the existence of the additional ETU2 process, and therefore the ratio I_547_/I_656_ decreases from 3.3 to 2.4 with the increase of the laser intensity. However, the slope of the green UC fluorescence is much larger than that of the red UC fluorescence for the shaped laser pulse because of the involvement of the additional ESA3 process, and thus the ratio I_547_/I_656_ increases from 1.0 to 1.8 with the increase of the laser intensity. Obviously, the green and red UC fluorescence tuning can also be obtained by varying the femtosecond laser intensity, which is the same as the previous study by controlling the continuous-wave (CW) laser intensity^32^, but this tuning range is much smaller than that by the spectral phase modulation (see Fig. 3). Since increasing the laser intensity will decrease the intensity ratio I_547_/I_656_ for the unshaped femtosecond laser pulse, while will increase it for the shaped femtosecond laser pulse, the experimental observation in Fig. 3 that the tuning range of the green and red UC fluorescence in lower laser intensity is larger than that in higher laser intensity by the square phase modulation can be well explained.

As shown in Fig. 3, the square phase modulation has shown to be an excellent method to realize the green and red UC fluorescence tuning in Er^3+^-doped NaYF_4_ nanocrystals. The basic physical control mechanism is that the multiple subpulse formation by the square phase modulation can create the additional ESA process (see Fig. 4), which results in the different excitation pathways for the green and red UC fluorescence generation. In our experiment, we only control the time separation of these subpulses by varying the modulation time Г. If the relative intensity in each subpulse can also be further manipulated by rationally designing the laser spectral phase, the tuning range of the green and red UC fluorescence will be further increased. Similarly, the purple (or blue) and red UC fluorescence tuning can also be realized by this spectral phase modulation since their control efficiencies are also different (see Figs 3(a) and 5(a)). The initial femtosecond laser pulse does not induce the ESA process due to the ultrashort pulse duration, but this situation can be changed by shaping the femtosecond laser pulse into multiple time-delayed subpulses (see Fig. 2(b,c)), the excited state population induced by one subpulse can be further pumped to the higher excited state by its subsequent subpulses. Since the lanthanide rare-earth ions, such as Yb^3+^, Ho^3+^, Dy^3+^, Nd^3+^, and so forth, have abundant energy levels and long excited state lifetime, the multiple time-delayed subpulses are easy to create the ESA process in these rare-earth ions, and therefore our proposed femtosecond pulse shaping technique can also be further extended to other lanthanide-doped nanomaterials.

## Conclusions

In summary, we have presented a new scheme to tune the UC fluorescence in lanthanide-doped nanocrystals by shaping the femtosecond laser pulse. Using a square phase modulation, we realized the green and red UC fluorescence tuning in Er^3+^-doped NaYF_4_ nanocrystals. Our experimental results showed that the shaped femtosecond laser pulse can affect the control efficiencies of the green and red UC fluorescence in different extent and therefore realize their tuning. Our analysis indicated that the multiple subpulse formation by the square phase modulation can create different excitation pathways for the green and red UC fluorescence generation, which results in their different control efficiencies. Furthermore, this physical control mechanism was experimentally validated by observing the effect of the laser intensity on the green and red UC fluorescence for the unshaped and shaped femtosecond laser pulses. These studies present a clear physical picture for the green and red UC fluorescence tuning in the Er^3+^-doped NaYF_4_ nanocrystals, which are very useful for further understanding and controlling the UC multicolor fine-tuning in lanthanide-doped nanocrystals, and are also expected to be applied in the multiplexed biolabeling.

## Additional Information

**How to cite this article**: Zhang, S. *et al.* Realizing up-conversion fluorescence tuning in lanthanide-doped nanocrystals by femtosecond pulse shaping method. *Sci. Rep.* **5**, 13337; doi: 10.1038/srep13337 (2015).
